# Genome-wide CRISPR-Cas9 screening identifies CLK1 inhibition as a strategy to restore PARP inhibitor sensitivity via ERCC1 isoform switching

**DOI:** 10.1093/procel/pwaf091

**Published:** 2025-11-04

**Authors:** Chaohua Liu, Fei Xu, Yutuan Wu, Jiana Li, Mengdong Ni, Siyu Xia, Lihua Chen, Haiyun Zhao, Min Yu, Yuqi Zhou, Meiqin Zhang, Jiajia Li, Xiaohua Wu, Yan Huang, Tao Zhu, Xiaojun Chen

**Affiliations:** Department of Gynecologic Oncology, Fudan University Shanghai Cancer Center, Shanghai 200032, China; Department of Oncology, Shanghai Medical College, Fudan University, Shanghai 200032, China; Cancer Institute, Fudan University Shanghai Cancer Center, Shanghai 200032, China; Department of Oncology, Shanghai Medical College, Fudan University, Shanghai 200032, China; Department of Gynecological Oncology, Zhejiang Cancer Hospital, Hangzhou Institute of Medicine (HIM), Chinese Academy of Sciences, Hangzhou 310024, China; Department of Gynecologic Oncology, Fudan University Shanghai Cancer Center, Shanghai 200032, China; Department of Oncology, Shanghai Medical College, Fudan University, Shanghai 200032, China; Department of Gynecologic Oncology, Fudan University Shanghai Cancer Center, Shanghai 200032, China; Department of Oncology, Shanghai Medical College, Fudan University, Shanghai 200032, China; Department of Gynecologic Oncology, Fudan University Shanghai Cancer Center, Shanghai 200032, China; Department of Oncology, Shanghai Medical College, Fudan University, Shanghai 200032, China; Department of Gynecologic Oncology, Fudan University Shanghai Cancer Center, Shanghai 200032, China; Department of Oncology, Shanghai Medical College, Fudan University, Shanghai 200032, China; Department of Gynecologic Oncology, Fudan University Shanghai Cancer Center, Shanghai 200032, China; Department of Oncology, Shanghai Medical College, Fudan University, Shanghai 200032, China; Department of Gynecologic Oncology, Fudan University Shanghai Cancer Center, Shanghai 200032, China; Department of Oncology, Shanghai Medical College, Fudan University, Shanghai 200032, China; Department of Gynecologic Oncology, Fudan University Shanghai Cancer Center, Shanghai 200032, China; Department of Oncology, Shanghai Medical College, Fudan University, Shanghai 200032, China; Department of Gynecologic Oncology, Fudan University Shanghai Cancer Center, Shanghai 200032, China; Department of Oncology, Shanghai Medical College, Fudan University, Shanghai 200032, China; Department of Gynecologic Oncology, Fudan University Shanghai Cancer Center, Shanghai 200032, China; Department of Oncology, Shanghai Medical College, Fudan University, Shanghai 200032, China; Department of Gynecologic Oncology, Fudan University Shanghai Cancer Center, Shanghai 200032, China; Department of Oncology, Shanghai Medical College, Fudan University, Shanghai 200032, China; Department of Gynecologic Oncology, Fudan University Shanghai Cancer Center, Shanghai 200032, China; Department of Oncology, Shanghai Medical College, Fudan University, Shanghai 200032, China; Department of Gynecological Oncology, Zhejiang Cancer Hospital, Hangzhou Institute of Medicine (HIM), Chinese Academy of Sciences, Hangzhou 310024, China; Department of Gynecologic Oncology, Fudan University Shanghai Cancer Center, Shanghai 200032, China; Department of Oncology, Shanghai Medical College, Fudan University, Shanghai 200032, China

**Keywords:** Ovarian cancer, CRISPR-Cas9 screen, PARP inhibitor, CLK1

## Abstract

Epithelial ovarian cancer (EOC) is an aggressive malignancy with limited therapeutic options. Poly(ADP-ribose) polymerase inhibitors (PARPi) have shown remarkable efficacy, especially in BRCA-mutant patients, and are approved as maintenance therapy to prevent recurrence after initial response to chemotherapy. However, the development of PARPi resistance poses a major clinical challenge. This study utilized a whole-genome CRISPR-Cas9 genetic screening to identify genes associated with PARPi sensitivity upon knockout. Based on the screening and validated through further experiments, we confirmed that CLK1 knockdown is synthetically lethal with PARPi in ovarian cancer. The combination of the PARPi Olaparib and CLK1 inhibitor TG003 exhibited potent anti-proliferative effects both *in vitro* and *in vivo*. Mechanistically, CLK1 inhibition downregulated the functional ERCC1-202 isoform, resulting in enhanced DNA damage and apoptosis. Our findings reveal a novel mechanism underlying PARPi sensitivity and suggest that targeting CLK1 in combination with PARPi may represent a promising therapeutic strategy for PARPi-resistant ovarian cancer.

## Introduction

Epithelial ovarian cancer (EOC) is one of the most lethal gynecological cancers that poses a significant threat to women’s health on a global scale (Siegel et al., 2022). Standard first-line treatment involves cytoreductive surgery followed by platinum-based chemotherapy; however, most patients eventually develop chemoresistance (Ledermann et al., 2018). In recent years, targeted therapies using poly(ADP-ribose) polymerase (PARP) inhibitors (PARPi) have shown encouraging clinical efficacy, particularly in patients with BRCA1/2 mutations, resulting in a 70% reduction in the risk of disease progression (Ledermann et al., 2018; Moore et al., 2018). Several PARPi, including olaparib, rucaparib, and niraparib, have been approved by the Food and Drug Administration (FDA) as maintenance therapy following platinum-based chemotherapy, significantly prolonging progression-free survival (PFS) and ­chemotherapy-free intervals (Aoki and Chiyoda, 2018; Nag et al., 2022). Despite these advances, the development of primary and acquired PARPi resistance has become a major clinical obstacle.

PARPi resistance in clinical settings involves diverse mechanisms, including restoration of homologous recombination (HR) repair, replication fork stabilization, and alterations of drug target or its pathway (Biegala et al., 2021; Dias and Moser, 2021; Noordermeer and van Attikum, 2019; Slade, 2020). HR restoration—particularly through BRCA1/2 re-expression via reversion mutations or epigenetic changes—represents the most well-characterized resistance pathway (Domchek, 2017; Norquist et al., 2011). Additional mechanisms such as upregulation of drug efflux pumps, PARP1 mutations, or loss of poly(ADP-ribose) glycohydrolase (PARG) activity further contribute to resistance (Gogola et al., 2018; Liu et al., 2024; Pettitt et al., 2018; Vaidyanathan et al., 2016). While these findings underscore the complexity of PARPi resistance, most mechanisms remain difficult to target clinically, highlighting the need for novel therapeutic strategies.

To identify promising candidate genes associated with PARPi sensitivity and to explore potential strategies for overcoming or delaying PARPi resistance, we performed a whole-genome CRISPR-Cas9 loss-of-function screen (­Sanjana et al., 2014; Shalem et al., 2014). This was followed by a kinase screen and functional validation, which led to the identification of CDC-like kinase 1 (CLK1) as a druggable vulnerability in PARPi-resistant ovarian cancer (OC). CLK1 belongs to the cdc2-like kinase family, which is known to be upregulated in various cancers, including gliomas and renal tumors (Colwill et al., 1996; Czubaty and Piekielko-­Witkowska, 2017). CLK1 plays a critical role in protein phosphorylation and alternative splicing regulation (Colwill et al., 1996). Previous studies have linked high CLK1 expression to poor prognosis in pancreatic cancer, where it promotes tumor progression through interaction with SRSF5, modulating splicing of genes such as METTL14 and cyclin L2 (Chen et al., 2021). CLK1 has also been reported to play a critical role in the regulation of splicing process in gastric cancer, where it was validated as a potential therapeutic target (Babu et al., 2020). In OC, CLK1 is involved in regulating the splicing process through its interaction with SPF45, affecting cell viability, proliferation, invasion, and migration (Duan et al., 2008; Liu et al., 2013). These findings suggest that CLK1 may be a promising therapeutic target in multiple cancers, yet its role in PARPi resistance remains unexplored.

Here, we identified CLK1 as one of the most promising candidate targets for overcoming PARPi resistance through genetic screening coupled with a kinase screening. We demonstrate that CLK1 inhibition can alleviate PARPi resistance in OC. Mechanistically, CLK1 regulates sensitivity to PARPi by mediating exon skipping of the ERCC1 gene. ERCC1 plays a key role in DNA repair pathways, reflecting its involvement in the DNA damage response (DDR) ­(Friboulet et al., 2013b; Zhang et al., 2007). Furthermore, we investigate potential therapeutic strategies for PARPi-­resistant OC and demonstrate that both genetic and ­pharmacological inhibition of CLK1 can overcome PARPi resistance. Collectively, our findings uncover a previously uncharacterized mechanism by which CLK1 regulates DNA damage repair and provide evidence that CLK1 inhibition may represent a potential therapeutic strategy for a subset of PARPi-resistant OCs.

## Results

### Genome-wide CRISPR-Cas9 knockout screen identifies genes linked to PARPi resistance

**Figure 1.**
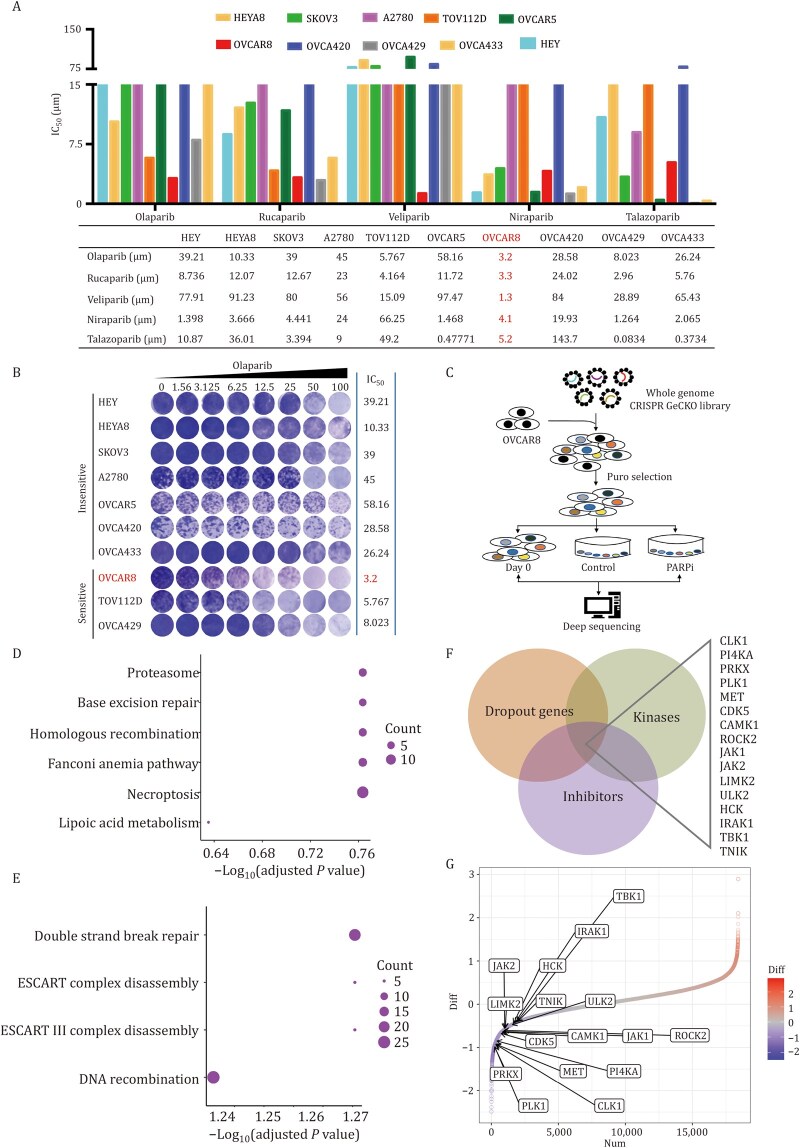
**Genome-wide CRISPR-Cas9 knockout screen identifies genes linked to PARPi resistance**. (A and B) IC_50_ values (µmol/L) of five PARPis in 10 ovarian cancer cell lines (A) and IC_50_ values (µmol/L) of Olaparib in 10 ovarian cancer cell lines were detected by CCK8 and displayed independently (B). (C) Schematic outline of the synthetic lethal screen. OVCAR8 cells transduced with a whole-genome sgRNA library and cultured with DMSO (control) or 2 µmol/L Olaparib (treated) for 2 weeks. sgRNAs from Day 0 and Day 14 were detected by PCR and quantified using next-generation sequencing. (D) KEGG analysis of negative screening genes. (E) Biological process (BP) analysis of negative screening genes. (F) Venn diagram of the dropout genes, genes with targeted inhibitors, and kinase genes. (G) Beta score gene differential analysis. The *x*-axis shows the beta score, and the *y*-axis shows the gene ranking.

To identify genes associated with PARPi resistance, we first tested the sensitivity of 10 OC cell lines to PARPi. Among them, the OVCAR8 cell line, which demonstrated sensitivity to various PARPi (Fig. 1A and 1B), was selected for the construction of a genome-wide CRISPR-Cas9 knockout library.

We performed a genome-wide CRISPR-Cas9 screen for dropout genes in the context of PARPi resistance. Using the GeCKOv2 sgRNA library, which targets 18,384 protein-­coding genes with 91,920 individual sgRNAs, we infected OVCAR8 cells at a multiplicity of infection (MOI) of 0.3. Selection for sgRNA and Cas9-expressing cells was carried out in the presence of puromycin for 2 days (Fig. 1C). The cells were harvested at two time points: immediately after puromycin selection (Day 0 group) and following 2 weeks of treatment with Olaparib or DMSO (dimethyl sulfoxide) (PARPi or Control group). Next-­generation sequencing was employed to quantify the abundance of all sgRNAs in these three cell populations at each time point, allowing us to calculate the essentiality scores for each gene using the MAGeCK algorithm (Li et al., 2014).

The MAGeCK-VISPR analysis pipeline included both data quality control and subsequent data analysis. Quality control steps involved sgRNA sequencing quality analysis, sgRNA reprint rate, and sgRNA abundance statistics, as well as the analysis of gene enrichment linked to cell growth. Data analysis included the review of sequencing data, sgRNA number statistics, identification of positively and negatively selected genes, and cluster analysis among samples. The sequencing results indicated a uniform distribution of sgRNAs, with a reprint rate exceeding 70%. Less than 1% of sgRNAs were lost in the sample collected at Day 0, and the average sgRNA abundance was 0.1 (Fig. S1A–C). At the same time, sgRNA knockout efficiency was reflected by the enrichment of ribosome pathway genes (Fig. S1D), confirming the successful construction of the library and the feasibility of the screening process.

The MAGeCK algorithm was used to analyze the differential selection of genes in the library screening results. Gene clusters with beta scores less than 0 correspond to dropout genes, the knockout of which leads to increased sensitivity to PARPi, promoting cancer cell death. Conversely, gene clusters with beta scores greater than 0 represent positively selected genes, where knockout confers increased resistance to PARPi (Fig. S1E; Table S1). In nine-square cluster analysis, the genes in Group 3 were identified as negative screening genes (Fig. S1F; Table S1). Kyoto Encyclopedia of Genes and Genomes and BP ­(biological process) pathway analyses of the Group 3 gene cluster revealed a strong association with DNA damage repair pathways (Fig. 1D and 1E; Table S1). Notably, our screen successfully recapitulated many known modulators of PARP sensitivity, including strong resistance with PARP1, PARP2, TP53BP1, and PARG depletion and sensitization following the depletion of XRCC1, RAD51AP2, FANCA, and FANCI (Fig. S1F) (Dev et al., 2018; Fang et al., 2019; Hewitt et al., 2021; Tsujino et al., 2023), validating its robustness.

To prioritize clinically actionable targets, we cross-referenced dropout genes from our CRISPR screen with kinases possessing clinically available inhibitors, identifying 16 high-confidence candidates (Fig. 1F). Beta scores quantifying their PARPi-sensitizing effects are shown in Fig. 1G.

### Kinase drug screening reveals CLK1 inhibition as a sensitizer of Olaparib in ovarian cancer

**Figure 2.**
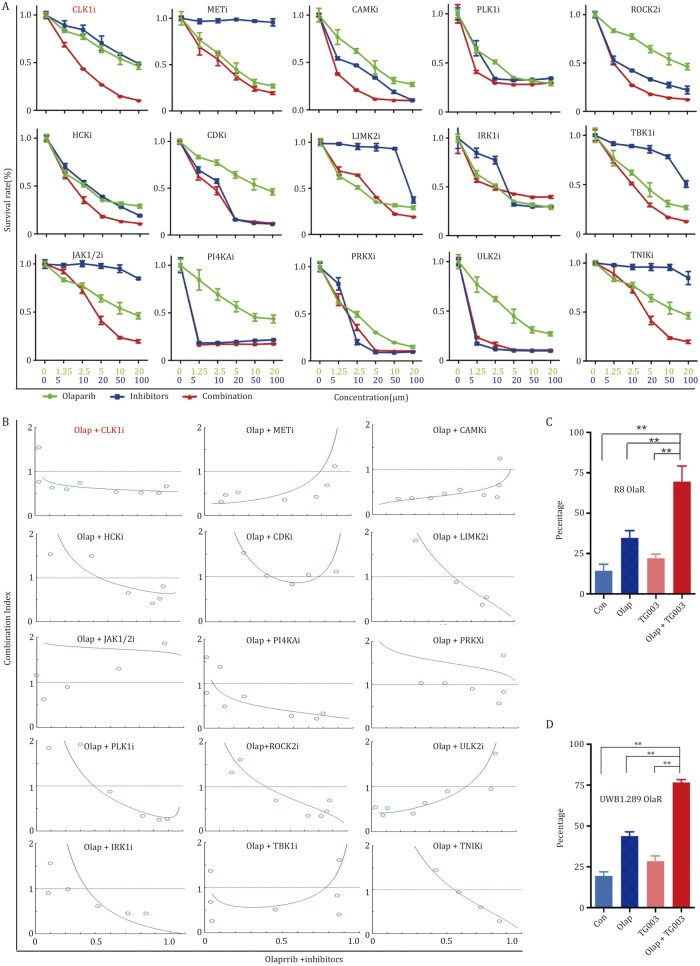
**Kinase drug screening reveals CLK1 inhibition as a sensitizer of Olaparib in ovarian cancer**. (A) CCK8 assays performed in R8 OlaR cells to assess the synergistic effect between PARPi Olaparib and 16 kinase inhibitors. (B) CI calculation using Compusyn software for the 16 kinase inhibitors combined with PARPi. CI < 1indicates synergism, CI = 1 indicates additive effects, and CI > 1 ­indicates antagonism. (C and D) Flow cytometry assay was performed to detect cell apoptosis in indicated cells treated with Olaparib (R8 OlaR, 20 µmol/L; UWB1.289 OlaR, 10 µmol/L) for 24 h.

To identify promising target genes among the 16 kinase genes (Fig. 1F and 1G), we induced drug resistance using an *in vitro* low-concentration gradient method, and successfully constructed OVCAR8 Olaparib-resistant cell line (R8 OlaR, BRCA1/2 wild-type) and UWB1.289 Olaparib-­resistant cell line (UWB1.289 OlaR, BRCA1 deletion type) (Fig. S2A). These cell lines were treated with inhibitors targeting the identified kinases in combination with Olaparib. The synergistic lethality of the drug combinations and the combination index (CI) were assessed by CCK8 cell proliferation assay and Compusyn software. The results showed that the combination of the CLK1 inhibitor TG003 and Olaparib significantly inhibited proliferation of tumor cells, with a CI value below 1 (Figs. 2A, 2B, and S2B), indicating that inhibition of CLK1 notably reversed Olaparib resistance and exhibited a strong synergistic effect.

To further confirm that CLK1 inhibition enhances the sensitivity of OC cells to Olaparib, we treated R8 OlaR and UWB1.289 OlaR with Olaparib, TG003 alone, or in combination for 24 h. While treatment with either Olaparib or TG003 alone had minimal effects on apoptosis, the combination of these two drugs significantly increased apoptosis in Olaparib-resistant cells (Fig. 2C and 2D). These findings suggest that inhibition of CLK1 effectively reverses Olaparib resistance and sensitizes OC cells to the drug.

### CLK1 regulates OC sensitivity to Olaparib and contributes to PARPi resistance

**Figure 3.**
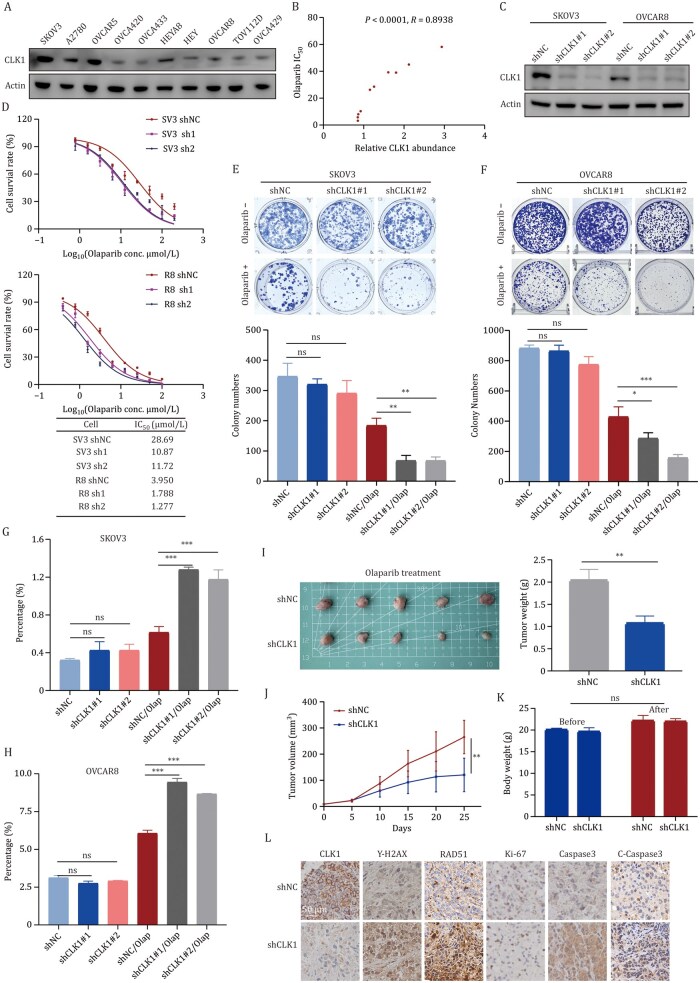
**CLK1 regulates OC sensitivity to Olaparib and contributes to PARPi resistance**. (A) Baseline expression of CLK1 protein in 10 ovarian cancer cell lines was examined by Western blot (WB). (B) Correlation analysis between CLK1 expression and sensitivity to Olaparib in ovarian cancer cell lines. (C) CLK1 knockdown was established in SKOV3 and OVCAR8 cells by stable transduction with shRNA targeting CLK1 (shCLK1), using non-targeting shRNA (shNC) as a control. CLK1 protein levels were assessed by WB. (D) Viability of shNC and shCLK1 SKOV3 and OVCAR8 cells following 96-h treatment with Olaparib, as determined by CCK-8 assay. (E and F) Clonogenic assay to evaluate colony formation efficiency in SKOV3 (E) and OVCAR8 (F) cells treated with Olaparib for 7–14 days (up). Quantification of colony number (down). (G and H) Flow cytometry assay was performed to detect cell apoptosis in SKOV3 (10 µm, 24 h) (G) and OVCAR8 (5 µm, 24 h) (H) cells with CLK1 stable knockdown. (I–K) *In vivo* evaluation of Olaparib efficacy after CLK1 knockdown. OVCAR8 and shCLK1 OVCAR8 cells (4 × 10^6^ cells) were subcutaneously injected into the left armpit of each mouse. When the tumor volumes reached approximately 50 mm^3^, the mice received an intraperitoneal injection of Olaparib (Olap, 50 mg/kg) three times a week. Three weeks post-injection, the mice were sacrificed, and the mouse body weights and tumor weight were quantified. Tumors from each group are shown in (I). Tumor growth curve (J) and nude mouse body weights of each group before and after administration (K) were quantified. (L) IHC detection of CLK1, γ-H2AX, RAD51, Ki-67, Caspase-3 and Cleaved-Caspase-3 expression.

Given that kinase drug screening revealed CLK1 as a promising candidate target, we detected the baseline expression levels of CLK1 in various OC cell lines (Fig. 3A) and observed a positive correlation between CLK1 protein abundance and sensitivity to Olaparib in these cell lines (Fig. 3B). Furthermore, an increase in CLK1 protein expression was noted in PARPi-resistant cells (Fig. S3A), which is consistent with previous findings suggesting that CLK1 mediates OC sensitivity to PARPi.

To investigate the role of CLK1 in Olaparib sensitivity, we constructed CLK1 stably knockdown and overexpression cell lines in both high-expressing SKOV3 and low-­expressing OVCAR8 cancer cell lines (Figs. 3C and S3B). CCK8 assays revealed that downregulation of CLK1 significantly enhanced the sensitivity of OC cells to Olaparib (Fig. 3D). By contrast, cells with CLK1 overexpression showed increased cell viability following Olaparib treatment (Fig. S3C). Correspondingly, cells with CLK1 knockdown exhibited a dramatic reduction in colony formation ability following Olaparib treatment (Fig. 3E and 3F). In contrast, cells with CLK1 overexpression exhibited the opposite result (Fig. S3D and S3E). In addition, flow ­cytometry analysis confirmed that knocking down CLK1 enhanced Olaparib-induced apoptosis (Figs. 3G, 3H, and S3F), whereas CLK1 overexpression reduced it (Fig. S3G–I). Western blot (WB) analysis further revealed that CLK1 knockdown alone did not affect Caspase-3 cleavage, but markedly potentiated Olaparib-induced cleavage of Caspase-3 (Fig. S3J), consistent with the apoptosis phenotypes observed by flow cytometry (Figs. 3G, 3H, and S3F).

Next, to assess the impact of CLK1 loss on tumor response to Olaparib treatment *in vivo*, we established xenograft models in BALB/c nude mice by subcutaneously injecting OVCAR8 with stable CLK1 knockdown (shCLK1) or control vectors. After tumor formation, Olaparib (50 mg/kg) was administered via intraperitoneal injection for 3 weeks. As shown in Fig. 3I and 3J, tumors in the shCLK1 group exhibited significantly reduced weight and volume compared to those in the control group, and the mouse body weights of each group remained unchanged before and after administration (Fig. 3K). Furthermore, immunohistochemical (IHC) analysis revealed that shCLK1 tumors exhibited lower Ki-67 (a proliferation marker) expression, but higher levels of γ-H2AX and RAD51 (DNA damage markers), as well as Caspase-3 and cleaved Caspase-3 (apoptosis markers) (Fig. 3L).

Finally, we examined CLK1 expression levels by IHC staining in 138 clinical samples of high-grade serous ovarian carcinoma and performed a prognostic analysis (Fig. S4A). We found that higher CLK1 expression was associated with a reduction in overall survival (OS), but not PFS (Fig. S4B). Moreover, TCGA (The Cancer Genome Atlas) data analysis showed that elevated CLK1 expression in OC patients ­correlated with poorer OS and PFS (Fig. S4C). These findings suggest that CLK1 plays a critical role in regulating OC ­sensitivity to Olaparib, both *in vitro* and *in vivo*, and serves as an independent prognosticator for OS in OC patients.

### The knockdown or inhibition of CLK1 activates the DNA damage response pathway

**Figure 4.**
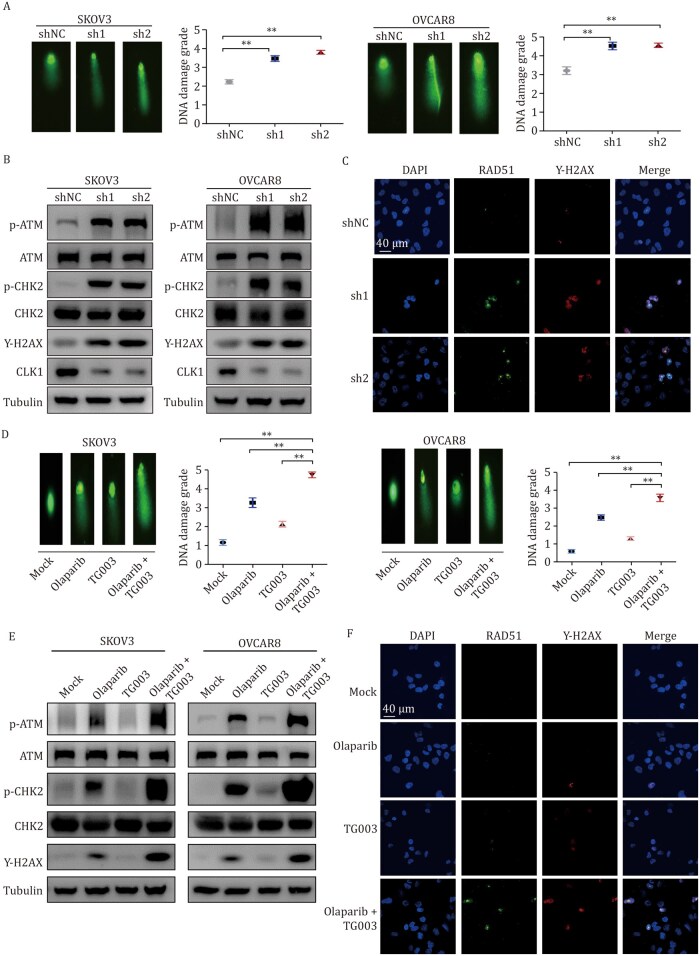
**The knockdown or inhibition of CLK1 activates the DNA damage response pathway**. (A) Comet assays conducted in SKOV3 and OVCAR8 cells with stable knockdown of CLK1 to assess DNA damage levels. (B) WB was conducted in SKOV3 and OVCAR8 cells with stable knockdown of CLK1 to detect the indicated antibodies. (C) The expression level of γ-H2AX and RAD51 detected by IF in OVCAR8 cells with stable knockdown of CLK1. (D) Comet assays conducted in SKOV3 and OVCAR8 cells treated with Olaparib (SKOV3: 10 μmol/L; OVCAR8: 5 μmol/L; for 24 h) and TG003 (10 μmol/L for 24 h) alone or in combination at low concentrations, respectively. (E and F) The expression level of γ-H2AX and other DNA damage markers detected by WB (E) and IF (F).

As DNA damage repairis a critical pathway involved in PARPi resistance (Noordermeer and van Attikum, 2019), which was also observed in the negative screening gene analysis (Fig. 1D and 1E), we next detected the DDR by comet assays in CLK1 knockdown OC cells. As shown in Fig. 4A, the nuclear tailing in CLK1 knockdown cells was significantly increased compared to the control group, indicating enhanced DNA damage. WB and immunofluorescence (IF) assays further confirmed that the expression of γ-H2AX and RAD51, key markers of double-strand DNA damage, was significantly elevated in the CLK1 stable knockdown cells (Fig. 4B and 4C).

In addition, DNA damage was detected in OC cells treated with Olaparib combined with TG003. As shown in Fig. 4D, the nuclear tailing was dramatically increased in the presence of co-treatment with CLKi and PARPi compared to CLKi or PARPi alone. Additionally, an increased expression of γ-H2AX was observed in the combination therapy group, as detected by both WB and IF (Fig. 4E and 4F). Furthermore, we demonstrated that CLK1 inhibition significantly impaired DNA HR repair efficiency (Fig. S5A). These findings suggest that CLK1 plays a role in the DDR pathway and mediates PARPi resistance, highlighting its potential as a therapeutic target.

### CLK1 is widely involved in the regulation of DNA damage repair-related proteins, especially ERCC1

**Figure 5.**
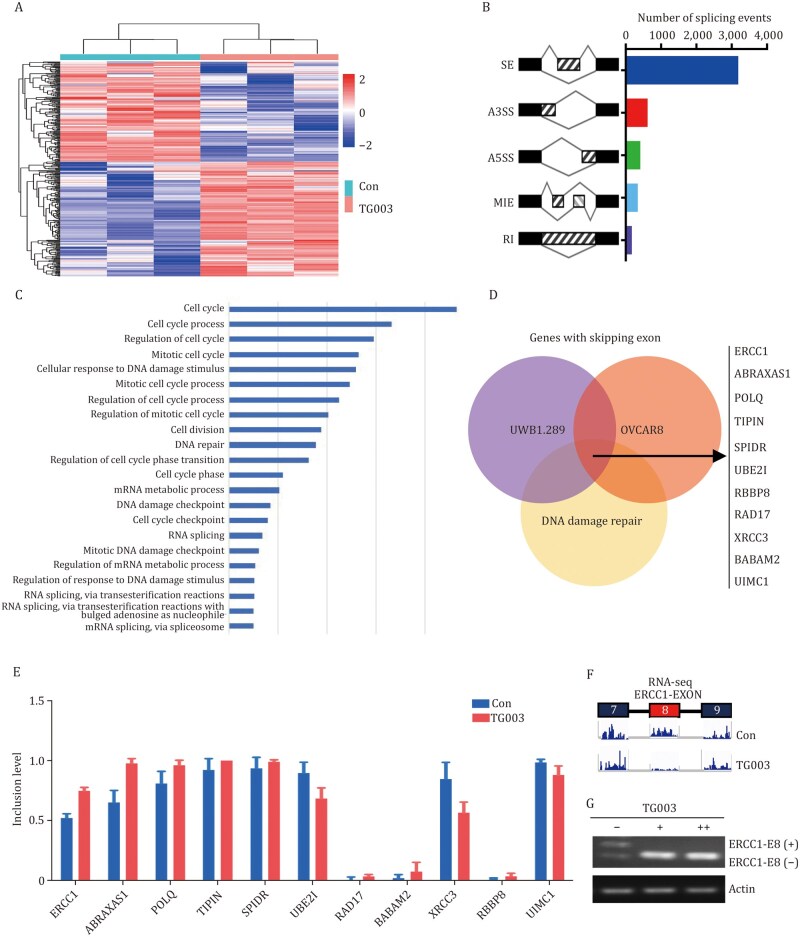
**CLK1 is widely involved in the regulation of DNA damage repair-related proteins, especially ERCC1**. (A) Heat map of differentially expressed genes in OVCAR8 cells following drug-induced suppression of CLK1. (B) Statistics of differential alternative splicing events in OVCAR8. (C) Gene ontology (GO) analysis of skipping exon genes in OVCAR8 cells. (D) Overlaps of the skipping exon genes in OVCAR8 and UWB1.289 and DNA damage repair-related genes. (E) Inclusion levels of the 11 skipping exon genes involved in DNA damage repair. (F) RNA sequencing peak map of ERCC1-EXON. (G) Detection of Exon 8 skipping event of the ERCC1 gene in OVCAR8 cells with increasing TG003 drug concentration.

To investigate the mechanism by which CLK1 mediates PARPi resistance, we treated R8 OlaR and UWB1.289 OlaR cells with 10 µmol/L CLK1 inhibitor TG003 and harvested cells for transcriptome sequencing 24 h later (Fig. 5A). Since CLK1 mainly affects gene expression by influencing mRNA alternative splicing process in eukaryotes, we focused on differential alternative splicing analysis using rMATS software. The analysis revealed that exon skipping (skipped exon [SE]) was the most significant splicing event (Fig. 5B).

Gene ontology (GO) analysis of these genes with differential exon skipping showed that they were predominantly associated with cell cycle, DNA damage repair, and mRNA alternative splicing processes (Fig. 5C). These findings are consistent with our previous observations suggesting that CLK1 affect DNA damage repair (Fig. 4). Based on these results, we hypothesized that CLK1 can regulate alternative splicing of mRNA involved in the process of DNA damage repair.

To further ascertain the specific downstream genes regulated by CLK1 through alternative splicing, we intersected the genes with differential exon skipping in both OVCAR8 and UWB1.289 cells with known DDR genes. This analysis identified 11 candidate’s genes (Fig. 5D), including ERCC1, ABRAXAS1, POLQ, TIPIN, SPIDR, UBE2I, RAD17, BABAM2, XRCC3, RBBP8 and UIMC1. As shown in Fig. 5E, the inclusion level values, which measure the extent of exon skipping for each gene, and the false discovery rate (FDR) values for the SE positions are displayed in Fig. S6A. Among these genes, ERCC1 (excision repair cross-complementation Group 1) showed the most pronounced exon skipping, making it a strong candidate for further investigation.

Integrative genomics viewer (IGV) analysis revealed that the eighth exon of the ERCC1 gene was significantly skipped (Fig. 5F). Moreover, increasing the concentration of TG003 led to more pronounced exon skipping of the ERCC1 Exon 8 (Fig. 5G), further confirming our sequencing data.

### CLK1/SRSF5 mediates exon skipping of the ERCC1 gene and contributes to OC sensitivity to PARPi

**Figure 6.**
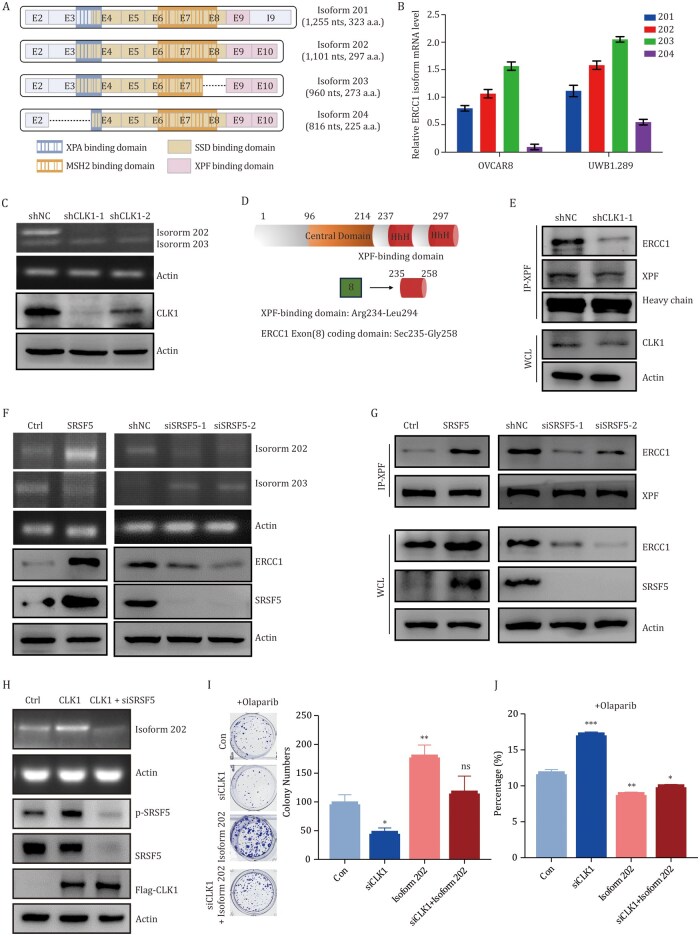
**CLK1/SRSF5 mediates exon skipping of the ERCC1 gene and contributes to OC sensitivity to PARPi**. (A) Schematic diagram of the main subtypes of the ERCC1 gene. (B) Expression levels of these four subtypes detected in OVCAR8 and UWB1.289 cell lines. (C) Expression level of ERCC1-202 and ERCC1-203 subtypes detected in shCLK1 OVCAR8 cells. (D) Schematic diagram of the wild-type long ERCC1-202 protein. The ERCC1 residues Arg234 to Leu294 constitute the XPF binding domain. Exon 8 of ERCC1 encodes Sec235 to Gly258. (E) Co-immunoprecipitation (Co-IP) analysis detecting the interaction between XPF and the ERCC1-202 isoform in OVCAR8 cells upon CLK1 knockdown. (F) Expression level of ERCC1-202 and ERCC1-203 isoforms after SRSF5 overexpression or knockdown in OVCAR8 cells. (G) Co-IP analysis detecting the interaction between XPF and the ERCC1-202 isoform after SRSF5 overexpression or knockdown in OVCAR8 cells. (H) Expression level of ERCC1-202 isoform after CLK1 overexpression and SRSF5 knockdown in OVCAR8 cells. (I) Clonogenic assay conducted in OVCAR8 cells with indicated treatments. (J) Flow cytometry assay detecting apoptosis ratios in OVCAR8 cells with indicated treatments.

The ERCC1 gene plays an important role in DNA damage repair, mainly involved in the nucleotide excision repair (NER) process and the HR repair process (Erdemir Sayan et al., 2024; Zhang et al., 2007). ERCC1 exists in four isoforms, including ERCC1-201, ERCC1-202, ERCC1-203, and ERCC1-204 (Fig. 6A). We first detected the expression levels of these subtypes in two ovarian cell lines, OVCAR8 and UWB1.289, and found that ERCC1-202 and ERCC1-203 were the most prominently expressed in these cell lines (Fig. 6B). After inhibiting the expression of CLK1, we observed a decrease in ERCC1-202 expression (Fig. 6C).

ERCC1 forms a complex with the XPF (xeroderma pigmentosum group F) endonuclease to repair DNA damage. When ERCC1 participates in the process of DNA damage repair, it mainly forms a heterodimer with XPF endonuclease, which catalyzes the generation of 5′ nicks at the DNA damage. The ERCC1-202 isoform is the only functional form that can bind to XPF, facilitating the formation of the ERCC1-XPF heterodimer and maintaining XPF stability (Friboulet et al., 2013a, 2013b). Since the amino acids encoded by Exon 8 of ERCC1 are critical for its interaction with XPF (Choi et al., 2005), we investigated whether CLK1 inhibition disrupts this interaction. As shown in Figs. 6D, 6E, and S7A, the binding of the ERCC1-202 isoform to XPF was significantly reduced upon CLK1 knockdown or ­pharmacological inhibition, indicating a reduction in the functional ERCC1-XPF complex. These findings support the hypothesis that CLK1 regulates ERCC1-202 isoform expression through alternative splicing, thereby affecting the process of DNA damage repair.

CLK1 is known to regulate alternative splicing, mainly by regulating the alternative splicing-related SR protein family proteins, including SRSF5 (Chen et al., 2021). Here, we confirmed the interaction between SRSF5 and CLK1 through co-immunoprecipitation (Co-IP) (Fig. S7B and S7C). The phosphorylation of SRSF5 was also inhibited in CLK1 knockdown cells (Fig. S7D and S7E). We propose that the CLK1/SRSF5 pathway induces aberrant exon skipping in ERCC1. To validate this, we assessed the effect of SRSF5 on ERCC1 expression. Overexpression of SRSF5 led to an increase in ERCC1-202 isoform expression, while knockdown of SRSF5 resulted in the opposite result (Figs. 6F, S7F, and S7G). In addition, in SRSF5 overexpressed cells, the interaction between XPF and ERCC1-202 was enhanced, while this interaction was impaired in SRSF5 knockdown cells (Fig. 6G). Importantly, SRSF5 knockdown also abolished the increased ERCC1-202 expression observed in CLK1-overexpressed cells (Figs. 6H and S7H), confirming that CLK1 mediates ERCC1 exon skipping through SRSF5.

To further validate the role of ERCC1-202 in PARPi resistance, we performed a rescue experiment by overexpressing ERCC1-202 in CLK1-knockdown cells, followed by Olaparib treatment. As shown in Fig. 6I and 6J, ERCC1-202 overexpression reversed the enhanced Olaparib sensitivity induced by CLK1 knockdown, as evidenced by restored colony-forming ability and reduced apoptosis. We next examined the expression of ERCC1-202, CLK1, and p-SRSF5 in PARPi-treated and PARPi-resistant cells. Elevated levels of all three proteins were observed specifically in PARPi-resistant cells (Fig. S7I and S7J). Conversely, treatment with the CLK1 inhibitor TG003—alone or in combination with Olaparib—led to decreased expression of ERCC1-202, CLK1, and p-SRSF5 (Fig. S7K). We also observed an upregulation of STING (stimulator of interferon genes) expression following TG003 treatment (Fig. S7L), a preliminary finding consistent with reports of ERCC1’s role in immunomodulation (Chabanon et al., 2019a), which merits further investigation beyond the scope of this study. Together, these results suggest that CLK1, through SRSF5-mediated alternative splicing of ERCC1, modulates OC sensitivity to PARPi.

### Targeted inhibition of CLK1 sensitizes OC to PARPi *in vivo*

**Figure 7.**
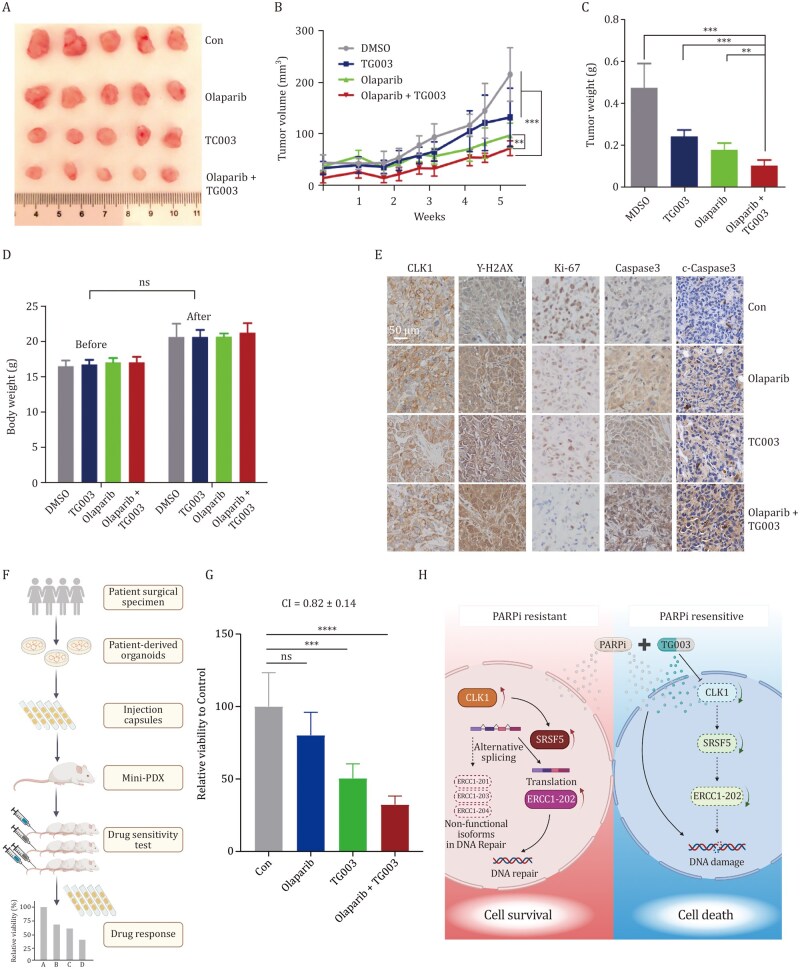
**Targeted inhibition of CLK1 sensitizes OC to PARPi *in vivo***. (A) R8 OlaR cells (4 × 10^6^) were subcutaneously injected into the left flank of each nude mouse. When the tumor volumes reached approximately 50 mm^3^, the mice were randomly assigned to four treatment groups (ctrl, Olaparib, TG003, Olaparib + TG003) and received intraperitoneal injections of Olaparib (100 mg/kg), TG003 (50 mg/kg), Olaparib + TG003 (100 mg/kg + 50 mg/kg), or DMSO, three times a week. Mice were sacrificed 3 weeks post-injection, and their body weights and tumor weights were measured. Tumors from each group are shown. (B and C) Tumor volume (B) and weight (C) of each group were measured. (D) Change in body weights of nude mice in each group before and after treatment (data are presented as mean ± SD; ns, *P *> 0.05; , **P < 0.01, ***P < 0.001; *n *= 5). (E) IHC staining of CLK1, γ-H2AX, Ki-67, Caspase-3, and cleaved Caspase-3 in xenograft tumor tissues. (F and G). Pharmacological testing of Olaparib and TG003 in mini-PDX models. Schematic diagram of the mini-PDX models (F). Quantitative analysis of drug response in mini-PDX models (G). (H) Schematic diagram depicting the molecular mechanism by which the CLK1-SRSF5-ERCC1 axis regulates PARPi-resistant ovarian cancer. In PARPi-resistant cells, elevated CLK1 expression promotes the expression of the functional ERCC1-202 isoform through SRSF5-mediated alternative splicing, enhancing DNA repair for cell survival, whereas the addition of the CLK1 inhibitor TG003 disrupts this splicing axis, downregulates ERCC1-202, impairs DNA repair, increases chromosomal aberrations, and ultimately resensitizes tumor cells to PARPi.

Building on our previous findings regarding the critical role of CLK1 in PARPi resistance at the cellular level, we next explored the clinical potential of targeting CLK1 to overcome PARPi resistance *in vivo*. We implanted the PARPi-resistant OC cell line R8 OlaR subcutaneously into nude mice. Once tumors had formed, the mice were assigned into four groups: a blank control group, TG003 or Olaparib single-drug group, and the combination group receiving both TG003 and Olaparib. As shown in Fig. 7A–C, the tumor growth rate in the single-drug groups was faster compared to the combination group. The tumor volume and weight were significantly lower in the combination group than in either single-drug groups. Importantly, the body weight of the nude mice in all groups remained stable throughout the experiment (Fig. 7D), indicating that the combination treatment was well-tolerated.

We further analyzed the expression of CLK1, γ-H2AX, Ki-67, Caspase-3, and cleaved Caspase-3 in tumor sections. The IHC staining showed that the Ki-67 expression was significantly lower in the combination group, and the expression levels of γ-H2AX, Caspase-3 and cleaved Caspase-3 were significantly higher in the combination group, indicating increased DNA damage and enhanced apoptosis (Fig. 7E). These findings indicate that the combination of Olaparib and TG003 reduces cell proliferation, exacerbates DNA damage, and promotes tumor cell apoptosis.

Further validation using mini-PDX (mini-patient-derived xenograft) models also supported these results, showing that TG003 could enhance the therapeutic effect of PARPi (Fig. 7F and 7G; Table S1). Together, these results provide compelling evidence that inhibition of CLK1 sensitizes OC to PARPi *in vivo*, and suggest that CLK1 inhibition could be a promising strategy for treating PARPi-resistant OC.

## Discussion

PARPi resistance remains a major clinical challenge in OC, limiting the long-term effectiveness of this treatment. Here, we demonstrate that CLK1 contributes to PARPi resistance by disrupting the alternative splicing of DDR genes, particularly ERCC1 (Fig. 7H). These findings position CLK1 not only as a potential predictive biomarker for ­disease progression but also as a promising therapeutic target for overcoming PARPi resistance in OC.

In this study, we adopted an unbiased, genome-wide CRISPR-Cas9 knockout screen in BRCA1/2-proficient OC cells to identify previously unknown genes whose loss has a profound impact on PARPi response. By combining genomic and functional data, this approach directly addresses the key challenge in selecting therapeutic agents with high specificity and minimal toxicity. Although our study was limited to the *in vitro* screening in tumor cell lines, our findings offer clinical insights into overcoming PARPi resistance and highlight potential therapeutic targets. In addition, other synthetic lethal candidates identified in our screen may provide further opportunities to explore effective targets or offer critical insights into the mechanisms of PARPi resistance in cancer. Notably, BRCA1 and BRCA2, whose loss was previously shown to provide PARPi sensitivity, did not emerge as significant hits in our screen (Table S1). A comparable observation has been documented in prior studies (Juhász and Smith, 2020; Tsujino et al., 2023). This is likely because acute inactivation of BRCA1/2 is lethal without adaptive mechanisms in cells (Hakem et al., 1997; Ludwig et al., 1997).

Recent studies have demonstrated the efficacy of PARPi in non-BRCA-mutated tumors, presumably through induction of PARP1-DNA trapping mechanisms (Saha et al., 2021). An abundance of knowledge has been built around resistance mechanisms in BRCA-mutated tumors (­Noordermeer and van Attikum, 2019). A recent study showed that in the HR-proficient background, Olaparib resistance was driven by overexpression of the multidrug resistance 1 gene (MDR1) (Christie et al., 2019). Additionally, in HRD (homologous recombination deficiency) cells, multiple heterogeneous co-existing mechanisms were found, including overexpression of MDR1, a decrease in PARP1 expression level, and partial reactivation of HR repair (­Chiappa et al., 2022). Our findings add to this growing body of work by demonstrating that CLK1 plays a significant role in mediating PARPi resistance in both BRCA1/2 wild-type (OVCAR8, SKOV3) and BRCA1-mutant (UWB1.289) OC cells. We observed that CLK1 abundance correlates with PARPi ­sensitivity, and its upregulation was found in Olaparib-­resistant cells. These results suggest that CLK1 regulates PARPi resistance independently of HR status, indicating that targeting CLK1 could potentially extend the use of PARPi beyond BRCA1/2-mutant tumors. Looking forward, our data raise the possibility that CLK1 may eventually inform predictive models for PARP inhibitor response, although this will require extensive future validation.

Our study identifies CLK1/SRSF5 as a critical regulator of functional isoform of ERCC1, which plays an essential role in DDR. CLK1 has previously been implicated in ­alternative splicing of genes governing cell cycle control (­Dominguez et al., 2016; Zhu et al., 2018), consistent with our sequencing data (Fig. 5A), showing that CLK1 knockdown preferentially induces exon skipping in cell cycle- and DDR-related genes. Notably, CLK1 promotes tumor progression in PDAC (pancreatic ductal adenocarcinoma) by phosphorylating SRSF5 to drive aberrant splicing of METTL14 and Cyclin L2 (Chen et al., 2021). Here, we expand these findings by identifying ERCC1 as a new target of the CLK1/SRSF5 axis in DNA damage repair, further highlighting the importance of CLK1 in maintaining genomic integrity. Given the pivotal role of CLK1 in PARPi resistance, we hypothesize that CLK1-mediated regulation of cell cycle progression and mRNA alternative splicing may critically influence PARPi efficacy—a mechanistic relationship ­warranting further exploration.

ERCC1, a core component of NER, partners with XPF to incise DNA 5′ to lesion sites (Matsunaga et al., 1995; Sijbers et al., 1996). Beyond NER, the ERCC1/XPF complex facilitates HR, double-strand break repair, and interstrand crosslink resolution (Zhang et al., 2007). *In vivo* studies using ERCC1-knockout mice reveal elevated endogenous DNA damage levels, supporting its role in HR regulation (Selfridge et al., 2001). ERCC1 expression also serves as a prognostic biomarker for cisplatin response in NSCLC (non-small cell lung cancer) and lung adenocarcinoma (Ganzinelli et al., 2021; Laufs et al., 2018). These established roles validate ERCC1 as a key downstream effector through which the CLK1/SRSF5 axis modulates PARPi sensitivity in OC.

Recent studies suggest that PARPi-induced tumor cell-intrinsic immune activation in NSCLC depends on ERCC1 deficiency, linked to its role in maintaining genomic stability (Chabanon et al., 2019a, 2019b). In our HR-proficient OC models, PARPi alone did not upregulate STING, whereas the combination with the CLK1 inhibitor TG003 increased STING expression (Fig. S7L). This suggests that CLK1 inhibition may mimic ERCC1 loss by disrupting DNA repair through altered ERCC1 splicing, potentially enhancing immunomodulatory signaling. This preliminary ­observation merits further study to explore whether CLK1 inhibition could augment PARPi efficacy via immune ­modulation in HR-proficient tumors.

While CLK1 has been implicated as a prognostic marker in multiple cancers (Lindberg and Meijer, 2021), our OC cohort showed significant OS but not PFS correlation, whereas TCGA data revealed both OS and PFS associations (Fig. S4). This discrepancy may reflect differences in cohort size or clinical characteristics, underscoring the need for validation in larger, well-annotated patient populations. We also sought to evaluate the protein-level interplay between CLK1 and the ERCC1-202 isoform in PARPi-sensitive versus resistant tumors. However, this effort faced two major constraints: first, the longitudinal acquisition of matched pre- and post-resistance clinical specimens is challenging, as many relapsed patients do not undergo repeat surgery; second, and more critically, there is a current lack of commercially available antibodies that can specifically detect the ERCC1-202 isoform without cross-reacting with other variants (Friboulet et al., 2013a). This technical limitation precludes reliable IHC assessment of ERCC1-202 in clinical samples at present and represents an important obstacle to translating ERCC1 isoform expression into a clinically usable biomarker for PARPi resistance.

In conclusion, our study establishes CLK1 as a key regulator of PARPi resistance in OC through its role in maintaining DNA damage repair fidelity. The synergistic effect observed with CLK1 inhibition and PARPi treatment not only reveals a promising combinatorial strategy to overcome resistance but also extends the potential of PARPi to HR-proficient tumors, thereby broadening the eligible patient population. Looking forward, components of the CLK1–SRSF5–ERCC1 axis may serve as predictive biomarkers to guide patient selection and optimize PARPi-based therapeutic strategies, pending the development of isoform-specific detection tools and validation in prospective clinical cohorts.

## Supplementary Material

pwaf091_Supplementary_Data

## Data Availability

The main data supporting the results in the study are available within the paper and its supplementary information. The raw and processed datasets generated during the ­current study are available for research purposes from the corresponding authors upon reasonable request.
