# Supplementary material for: Genome-wide CRISPR-Cas9 screening identifies CLK1 inhibition as a strategy to restore PARP inhibitor sensitivity via ERCC1 isoform switching
Source: Protein Cell. 2025 Nov 4;17(3):248–62. doi: 10.1093/procel/pwaf091 (PMC12987568; doi:10.1093/procel/pwaf091)
Supplement: pwaf091_Supplementary_Data [file pwaf091_supplementary_data.zip › TableS2.pdf]

Supplementary Table 2.

**Real-Time PCR Primers**

| Target Gene | Forward primer | Reverse primer |
|---|---|---|
| RT_Actin | CATGTACGTTGCTATCCAGGC | CTCCTTAATGTCACGCACGAT |
| RT_SRSF5 | AGTGGCTGTCGGGTATTCATC | CCGTCCATATCCCTTGAAGAATC |
| RT_201 | TCTCCTTCCCCCAACTCCTT | CCCTCCTGACCACATTTGGA |
| RT_202 | GGGCTCGTGCAGGACATC | CCTCCTGACCACATTTGGATCT |
| RT_203 | CATAAGGCCAGATCTTCTCTTGATG | CCAGCGGACCTCCTGATG |
| RT_204 | GGAATTACGTCGCCAAATTCC | ATACCCCTCGACGAGGATGA |

**sh/siRNA sequences**

| Target Gene | Sequence |
|---|---|
| CLK1-1 | CCGAGAAGCUCAACAACUA |
| CLK1-2 | GCUCGCUAUGGAGAAUUAC |
| SRSF5-1 | GGACCAAGTGTATGGTCAAGA |
| SRSF5-2 | GCCCTTTAAGCAGCTTCATAA |
