# Supplementary material for: Genome-wide CRISPR-Cas9 screening identifies CLK1 inhibition as a strategy to restore PARP inhibitor sensitivity via ERCC1 isoform switching
Source: Protein Cell. 2025 Nov 4;17(3):248–62. doi: 10.1093/procel/pwaf091 (PMC12987568; doi:10.1093/procel/pwaf091)
Supplement: pwaf091_Supplementary_Data [file pwaf091_supplementary_data.zip › PAC-25408-CXJ-Supplemental materials.pdf]

Materials and Methods

Cell culture and establishment of stable cell lines

Ovarian cancer cell lines used in this study, SKOV3, A2780, HEY, HEYA8, TOV112D, OVCAR5, OVCAR8, OVCA420, OVCA429 and OVCA433 were obtained from ATCC and cultured in Dulbecco's Modified Eagle Medium (DMEM, Gibco) with 10% fetal bovine serum (FBS, Gibco) and penicillin (100U/mL)/streptomycin (0.1 mg/mL) (15140-122, Gibco), and were negative for mycoplasma testing. The shRNAs specific for CLK1 (Table S2) were cloned into the pLKO.1 vector (Sigma-Aldrich, St louis, MO, USA). The stable cell lines were constructed as described(Xu et al., 2021).

Genome-wide CRISPR knock-out library screen

We used a CRISPR-Cas9 genome-wide knockout library. The library consisted of 91,920 sgRNA targets for 18,384 protein-coding genes, which were synthesized at CustomArray and amplified by PCR as previously described(Shalem et al., 2014, Jeselsohn et al., 2018). For lentiviral production, 293T cells were transfected with amplified GeCKOv2 plasmid DNA, psPAX2, and pMD2.G using X-tremeGENE HP DNA Transfection Reagent (Roche). Supernatant was collected and filtered 72 hours after transfection, and stored at −80°C. 1×10^8 cells were infected at a low MOI (0.3) to ensure that most cells received only one virus with high probability. After three days of puromycin selection, the surviving cells were divided into three replicates of 3×10^7 cells. One replicate was immediately collected as the day 0 cells, which served as controls to identify positively or negatively selected genes or pathways. The other two replicates were cultured with DMSO or 2μM Olaparib (PARPi, MCE, HY-1012) respectively for 14 days before genomic DNA extraction and library preparation. Amplified DNA was sequenced using Illumina sequencing technology.

CRISPR-screen data analysis

The CRISPR-screen data were analyzed by the MAGeCK and MAGeCK-VISPR algorithms as previously described(Li et al., 2015). MAGeCK-VISPR uses the ‘b score’ standard to perform the gene selection process. The meaning of ‘b score’ is similar to

the term of ‘log2 FC’ in other expression analysis, and b score>0 means the corresponding gene is positively selected, while b score<0 means negatively selected. The b score >0.5 or <-0.5 was considered significant. The MAGeCK-VISPR model takes gRNA read count as a negative binomial variable, and its mean value is determined by the linear combination of sample sequencing depth, gRNA efficiency, and gene b scores. Then MAGeCK-VISPR establishes a maximum likelihood (MLE) model to simulate all gRNA read counts of all samples, and uses an expectation maximization algorithm to iteratively estimate gRNA efficiency and gene b scores.

Drug Synergy Analysis

Small molecule inhibitors, TG003(CLKi, HY-15338), KDU691(PI4Kai, HY-12912), GSK690693(PRKXi, HY-10249), Capmatinib(METi, HY-13404), Roscovitine(CDK5i, HY-30237), TAK-733(CAMK1i, HY-13449), Ruxolitinib(JAK1/2i, HY-50856), KD025(ROCK2i, HY-15307), T56LIMKi(LIMK2i, HY-19352), MRT68921(ULK2i, HY-100006), IRAK-1-4 InhibitorI(IRAK1i, HY-13329), BI2536(PLK1i, HY-50698), PP2(HCKi, HY-13805), BX795(TBK1i, HY-10514), NCB-0846(TNIKi, HT-100830) were all purchased from MedChemExpres. For drug synergy studies, ovary cancer cells (3000/well) were seeded in 96 well plates for overnight incubation and treated with different doses of inhibitors for 5 days. Cell viability was evaluated by measuring the 450 nm absorbance with the Cell Counting Kit-8 (CCK-8) (YEASEN, Shanghai). Each concentration was tested in triplicate. The IC50 value was calculated and performed in GraphPad Prism v6.0. Drug synergistic effects were calculated based on the CompuSyn Software: software. CI < 1 indicates synergism, CI = 1 indicates additive effects, and CI > 1 indicates antagonism.

Cell apoptosis analysis

For cell apoptosis analysis, treated cells were washed with PBS, digested using trypsin, rinsed in PBS, and then resuspended in 1× binding buffer (YEASEN, Shanghai, China). Cells were incubated with fluorescein isothiocyanate (FITC)-Annexin V (5 μL) for 5 min and 7-AAD (10 μL) for 10 min in the dark at 4 °C. The mixture was further

analyzed with a BD Flow Cytometer and the FlowJo software.

Neutral comet assay

The neutral comet assay was performed as previously described(Swain and Subba Rao, 2011). Briefly, 1×10^5 cells were mixed with molten LMAgarose (Sangon Biotech;Shanghai, China) at room temperature at a ratio of 1:10 (v/v), and 50 µl of this mixture was immediately dispersed evenly onto the comet slide which was placed flat at 4°C in the dark for 1 hour, then immersed in pre-chilled lysis buffer for 2 hours. After lysis, the slides were washed with distilled water twice, soaked in a neutral electrophoresis buffer for 20 minutes, and then an electric field was applied. Then the cells were stained with Green-DNA Dye (A502040, Sangon Biotech; Shanghai, China) and photographed using an Olympus microscope with an attached camera. The scoring was performed as a grade of 0 to 4 according to tail size.

Quantitative reverse transcription and quantitative RT-PCR (RT-qPCR)

Total RNA was isolated using TRIzol reagent (Life Technologies, Waltham, MA, USA), and 1 µg of total RNA was used to prepare cDNA using the PrimeScript RT reagent Kit (Takara, Japan). RT-qPCR was performed in triplicate using TB Green Premix (Takara, Japan) on a LightCycler 480 Real-Time system (Roche). The primers for ERCC1 are referenced(Friboulet et al., 2013).

Western blotting (WB)

Cells were lysed in RIPA buffer with cocktails (YEASON, Shanghai, China). Cell lysates were extracted and loaded on an SDS-polyacrylamide gel and transferred onto PVDF membrane (Millipore, Billerica, MA, USA). The antibodies used for WB are listed as follows: anti-phospho-ATM (#5883, Cell Signaling Technology ,Danvers, Massachusetts, USA); anti-phospho-CHK2(#2661, Cell Signaling Technology ,Danvers, Massachusetts, USA); anti-γ-H2AX (#2577, Cell Signaling Technology ,Danvers, Massachusetts, USA) ; anti-CLK1 (AB40759, AbSci, Baltimore, Maryland, USA); anti-ERCC1(sc-53281, Santa Cruz Biotechnology, Dallas, Texas,

USA); anti-XPF (sc-136153, Santa Cruz Biotechnology, Dallas, Texas, USA); anti-SRSF5(sc-57954, Santa Cruz Biotechnology, Dallas, Texas, USA) ; anti-Actin (66009-1-Ig, Proteintech); anti-GAPDH(#5174,Cell Signaling Technology, Danvers, Massachusetts, USA);anti-Ki-67(#9129, Cell Signaling Technology, Danvers, Massachusetts, USA) ;anti-Caspase3(#9662, Cell Signaling Technology ,Danvers, Massachusetts, USA)

Immunofluorescence

1×10^5 cells were seeded onto microscope cover glass in 24-well cell culture plates, and cultured overnight, and subsequently exposed to drugs for 24 h. Then cells were fixed with 4% paraformaldehyde, permeabilized with 0.5% Triton X-100, blocked with normal goat serum, and incubated with primary antibody and fluorescent secondary antibodies. Nuclei were stained with DAPI (Servicebio; Wuhai, China). Confocal microscopy images were obtained using Olympus FV1000.

Colony formation assay

2000 cells/well were plated in triplicate on a 6-well plate, incubated for 24 h, and treated with drugs for 2 weeks. Surviving colonies were fixed with 4% formalin and stained with 0.05% crystal violet (Servicebio; Wuhai, China), and the number of colonies (more than 50 cells) was counted.

Library preparing and RNA sequencing

Transcriptome sequencing was performed by OE Biotech Co., Ltd (Shanghai, China). Total RNA was extracted using the mirVana miRNA Isolation Kit (Ambion) following the manufacturer’s protocol. RNA integrity was evaluated using the Agilent 2100 Bioanalyzer (Agilent Technologies, Santa Clara, CA, USA). The samples with RNA Integrity Number (RIN) ≥ 7 were subjected to the subsequent analysis. The libraries were constructed using TruSeq Stranded mRNA LTSample Prep Kit (Illumina, San Diego, CA, USA) according to the manufacturer’s instructions. Then these libraries were sequenced on the Illumina sequencing platform (HiSeqTM 2500 or Illumina

HiSeq XTen) and 125bp/150bp paired-end reads were generated. FPKM value of each gene was calculated using cufflinks, and the read counts of each gene were obtained by htseq-count. DEGs were identified using the DESeq (2012) R package functions estimateSizeFactors and nbinomTest. P-value < 0.05 and foldChange >2 or foldChange < 0.5 was set as the threshold for significantly differential expression. Hierarchical cluster analysis of DEGs was performed to explore genes expression pattern. GO enrichment and KEGG pathway enrichment analysis of DEGs were respectively performed using R based on the hypergeometric distribution. The alternatively splicing analysis of differentially regulated transcripts isoforms or exons was performed using rMATS software.

Co-Immunoprecipitation (Co-IP)

The Co-IP was performed as previously described(Xu et al., 2021). Briefly, the protein lysate was incubated with the beads-antibody complex and detected by IB.

Xenografts in nude mice

Ovarian cancer cells (4×10^6 cells per mouse) were subcutaneously inoculated into female BALB/C nude mice aged 5 weeks (Shanghai SLAC Laboratory Animal Co., Ltd; Shanghai, China). Tumor volumes were calculated by (Length × Width2) /2. When the mean tumor volumes reached $50mm^3$, mice were divided into 4 groups and received administration via intraperitoneal injection of Olaparib (100 mg/kg), TG003 (50 mg/kg), Olaparib + TG003, vehicle control three times a week for 5 weeks. The effect of drugs was evaluated by the tumor weight and the body weight of mice. Nude mice were housed in a pathogen-free environment in Department of Laboratory Animal Science in Shanghai Medical College of Fudan University. The animal experiment was performed according to the animal ethical principles.

Drug response test of mini patient-derived xenograft (mini-PDX) models

Drug efficacy was evaluated in vivo using mini-PDX models as previously described(Xiao et al., 2022). The tumor samples were collected from patients who had

undergone secondary cytoreductive surgery after developing resistance to PARPi (Olaparib or Niraparib) maintenance therapy. Briefly, fresh human ovarian tumor tissues were washed in HBSS buffer, and necrotic debris was removed. Single-cell suspensions were prepared using the MiniPDX™ sample processing system, followed by cell counting. The cells were resuspended and adjusted to an appropriate concentration, then loaded into OncoVee® capsules (LIDE Biotech, Shanghai, China), with each capsule containing approximately 2000 cells. The capsules were implanted subcutaneously into 5-week-old female BALB/c nude mice (five capsules per mouse). Drug treatment was initiated on the day of implantation. Mice were administered Olaparib (100 mg/kg) and TG003 (50 mg/kg), either alone or in combination, once daily for seven consecutive days. All drugs were dissolved in a vehicle consisting of DMSO, PEG300, and Tween-80. Control mice received an equal volume of vehicle (0.5% HPMC and 0.2% Tween-80 solution). After 7 days, capsules were retrieved, and cell viability was assessed by measuring ATP activity using the CellTiter-Glo® Luminescent Cell Viability Assay (Promega, Madison, WI, USA). The tumor cell growth inhibition rate was calculated according to the published formula.

Statistical analysis

Statistical analysis was performed using GraphPad Prism v6.0. Statistical significance was conducted by the two-tailed unpaired Student's t-test. Survival analyses were determined by Kaplan-Meier curve and log-rank test. All the data are shown as mean ± SD. P-values were demonstrated in the graphs using * for $P < 0.05$, ** for $P < 0.01$, and *** for $P < 0.001$. n.s. represents not significant.

FRIBOULET, L., OLAUSSEN, K. A., PIGNON, J. P., SHEPHERD, F. A., TSAO, M. S., GRAZIANO, S., KRATZKE, R., DOUILLARD, J. Y., SEYMOUR, L., PIRKER, R., FILIPITS, M., ANDR , F., SOLARY, E., PONSONNAILLES, F., ROBIN, A., STOCLIN, A., DORVAULT, N., COMMO, F., ADAM, J., VANHECKE, E., SAULNIER, P., THOMALE, J., LE CHEVALIER, T., DUNANT, A., ROUSSEAU, V., LE TEUFF, G., BRAMBILLA, E. & SORIA, J. C. 2013. ERCC1 isoform expression and DNA repair in non-small-cell lung cancer. *N Engl J Med,* 368**,** 1101-10.

JESELSOHN, R., BERGHOLZ, J. S., PUN, M., CORNWELL, M., LIU, W., NARDONE, A., XIAO, T., LI, W., QIU, X., BUCHWALTER, G., FEIGLIN, A., ABELL-HART, K., FEI, T., RAO, P., LONG, H.,

KWIATKOWSKI, N., ZHANG, T., GRAY, N., MELCHERS, D., HOUTMAN, R., LIU, X. S., COHEN, O., WAGLE, N., WINER, E. P., ZHAO, J. & BROWN, M. 2018. Allele-Specific Chromatin Recruitment and Therapeutic Vulnerabilities of ESR1 Activating Mutations. *Cancer Cell,* 33**,** 173-186 e5.

LI, W., KOSTER, J., XU, H., CHEN, C. H., XIAO, T., LIU, J. S., BROWN, M. & LIU, X. S. 2015. Quality control, modeling, and visualization of CRISPR screens with MAGeCK-VISPR. *Genome Biol,* 16**,** 281.

SHALEM, O., SANJANA, N. E., HARTENIAN, E., SHI, X., SCOTT, D. A., MIKKELSON, T., HECKL, D., EBERT, B. L., ROOT, D. E., DOENCH, J. G. & ZHANG, F. 2014. Genome-scale CRISPR-Cas9 knockout screening in human cells. *Science,* 343**,** 84-87.

SWAIN, U. & SUBBA RAO, K. 2011. Study of DNA damage via the comet assay and base excision repair activities in rat brain neurons and astrocytes during aging. *Mech Ageing Dev,* 132**,** 374-81.

XIAO, Y., MA, D., YANG, Y.-S., YANG, F., DING, J.-H., GONG, Y., JIANG, L., GE, L.-P., WU, S.-Y., YU, Q., ZHANG, Q., BERTUCCI, F., SUN, Q., HU, X., LI, D.-Q., SHAO, Z.-M. & JIANG, Y.-Z. 2022. Comprehensive metabolomics expands precision medicine for triple-negative breast cancer. *Cell Research,* 32**,** 477-490.

XU, F., LI, J., NI, M., CHENG, J., ZHAO, H., WANG, S., ZHOU, X. & WU, X. 2021. FBW7 suppresses ovarian cancer development by targeting the N(6)-methyladenosine binding protein YTHDF2. *Mol Cancer,* 20**,** 45.

Fig. S1 Genome-wide CRISPR-Cas9 knockout screen identifies genes linked to PARPi resistance. Related to Fig. 1

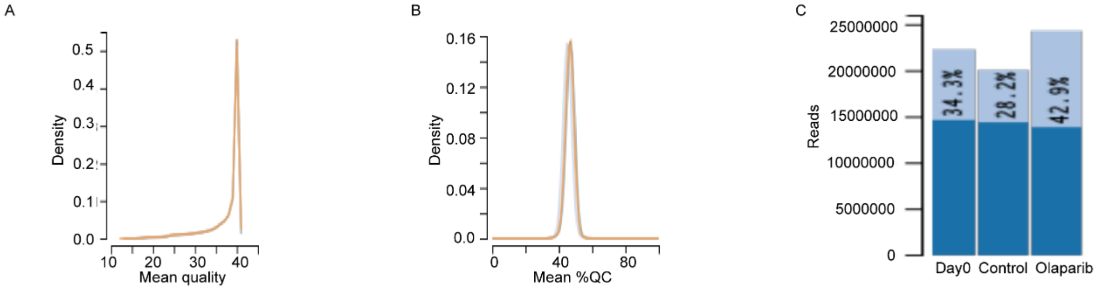

D

Ribosome gene enrichment analyses

Sample	NegSelQC	NegSelQCPval	NegSelQCPval Permutation	NegSelQCPval PermutationFDR	NegSelQCGene
Day0	0	1	1	1	0.0
Control	0.72189	2.3986e-41	0	0	86
Olaparib	0.71959	2.7109e-35	0	0	86

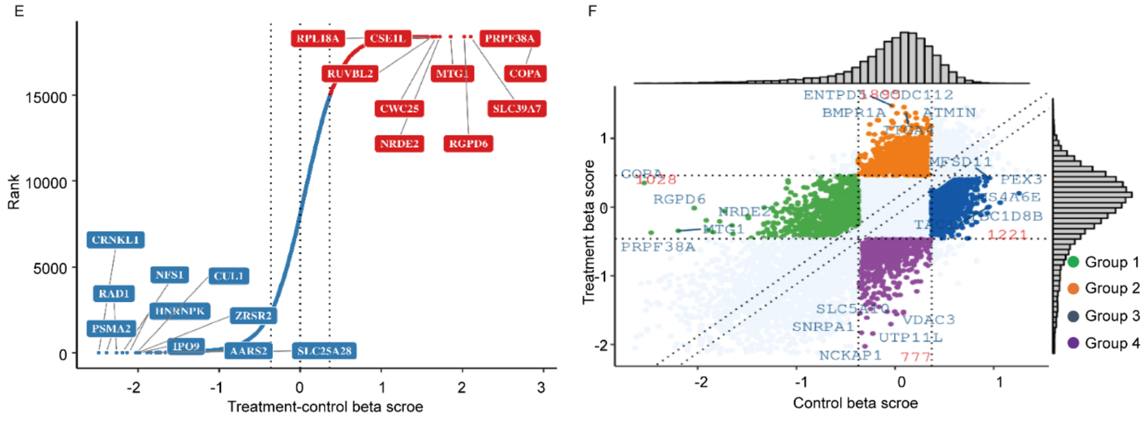

G

ENTREZID	Control	Treatment	Diff	Gene	Loss lead to Resistance/Sensitivity
142	-0.25407	1.2061	1.46017	PARP1	Resistance
10038	0.10094	1.02	0.91906	PARP2	Resistance
7158	0.13912	0.8124	0.67328	TP53BP1	Resistance
8505	0.19767	0.73581	0.53814	PARG	Resistance
7515	0.46601	-2.0202	-1.55419	XRCC1	Sensitivity
729475	0.57328	-0.59836	-1.17164	RAD51AP2	Sensitivity
2175	-0.31428	-0.86327	-0.54899	FANCA	Sensitivity
55215	-0.64301	-1.5675	-0.92449	FANCI	Sensitivity

A. sgRNA average distribution map. B. Average GC content map. C. sgRNA reprint rate. D. Ribosome gene enrichment analysis. E. Beta score gene differential analysis. The x-axis shows the beta score, and the y-axis shows the gene ranking. F. Differential gene nine-square cluster analysis. G. The list of screened known modulators of PARPi sensitivity or resistance.

Fig. S2 CLK1 inhibition sensitizes ovarian cancer to Olaparib. Related to Fig. 2

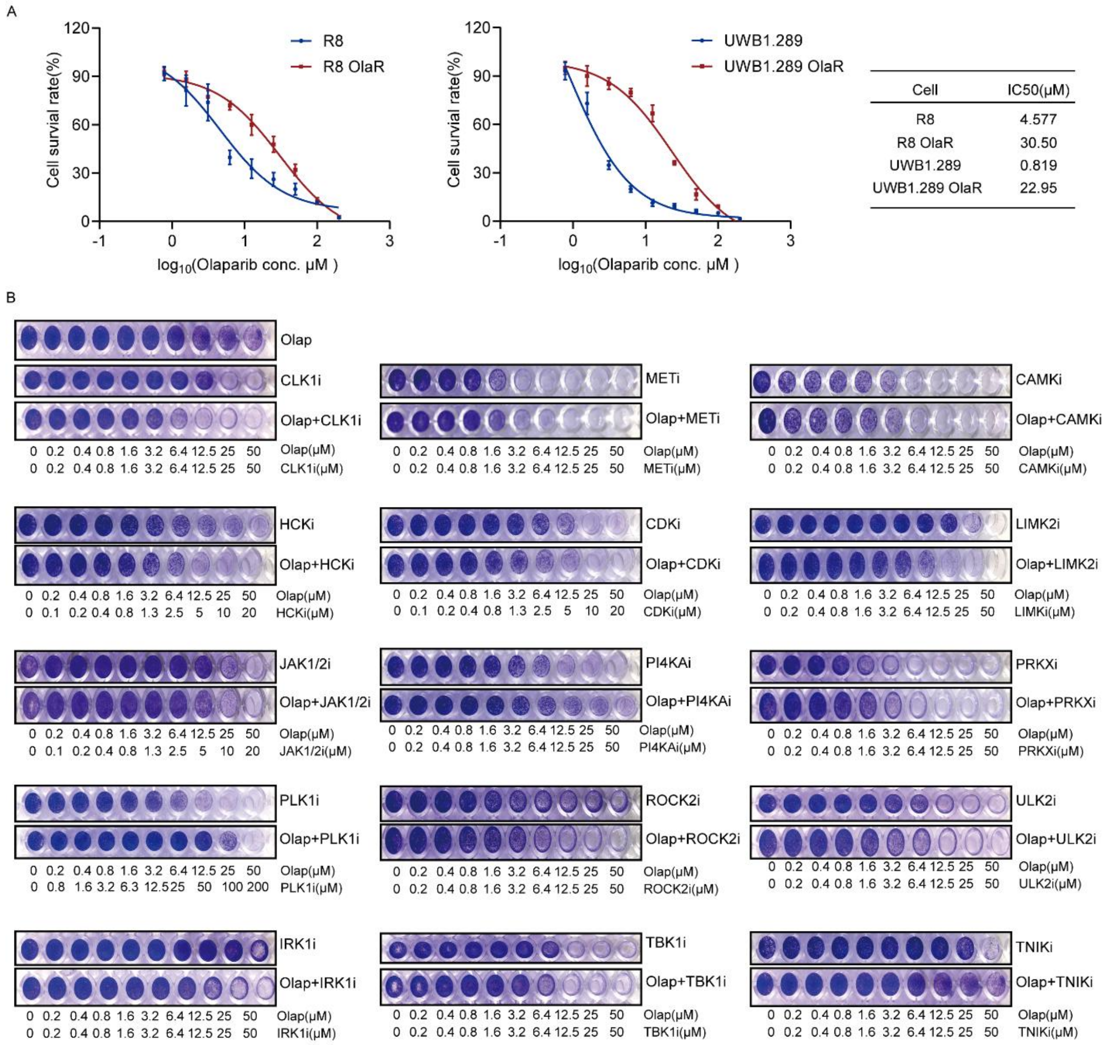

A. Construction of drug-resistant cell lines. The non-BRCA mutated cell line OVCAR8 and BRCA mutated cell line UVWB1.289 were subjected to gradually increasing concentration of Olaparib to allow for the development of acquired resistance. IC50 of Olaparib-resistant OVCAR8 and UWB1.289 (R8 OlaR, UWB1.289 OlaR) and parent original parent OVCAR8 (R8), UWB1.289 cell lines were detected by CCK8. B. CCK8 assays performed in R8 OlaR cells to assess the synergistic effect between PARPi Olaparib and 16 kinase inhibitors.

Fig. S3 CLK1 regulates OC sensitivity to Olaparib and contributes to PARPi resistance. Related to Fig. 3

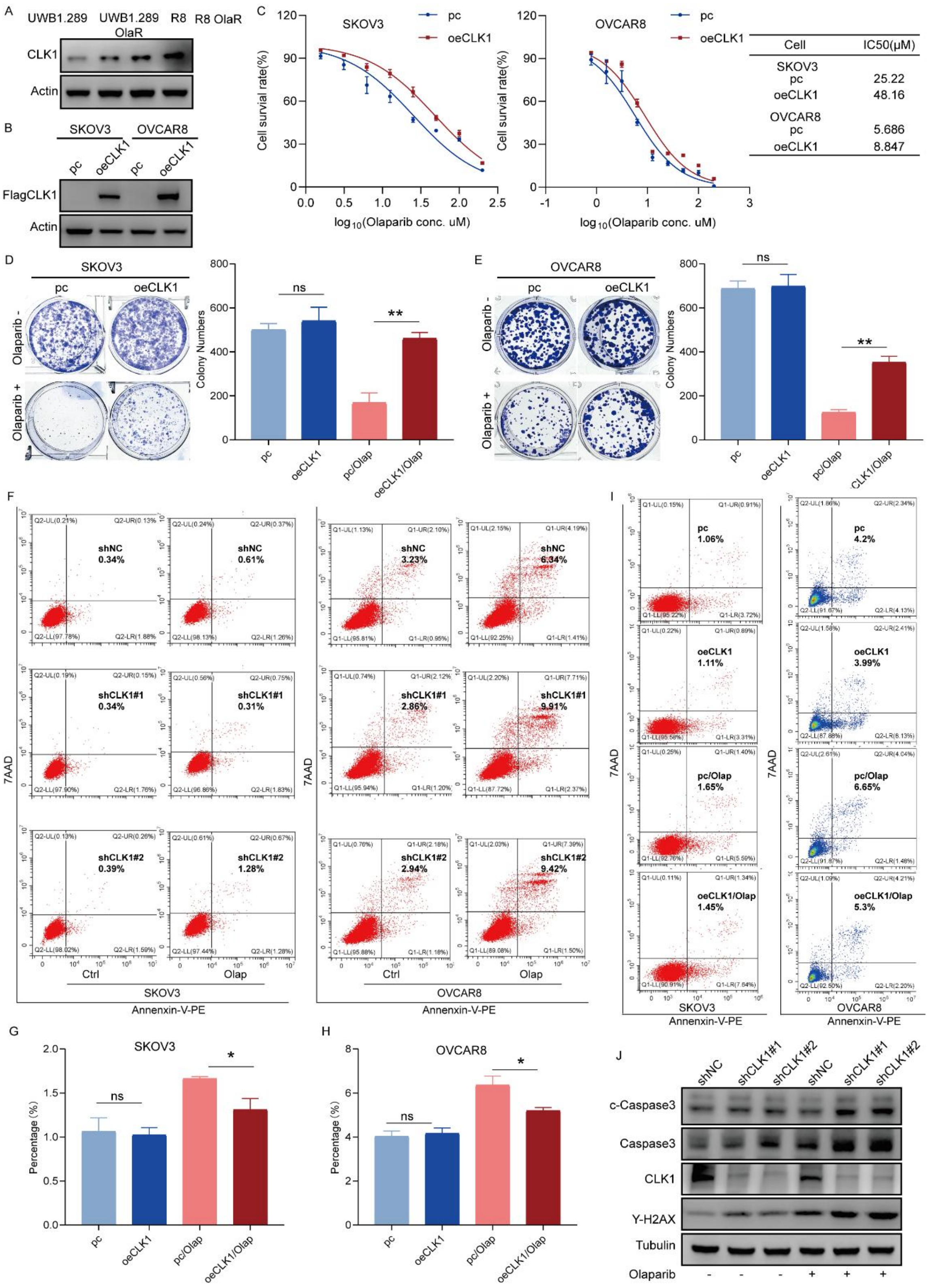

A. PARPi-resistant and its original parent OC cells were collected, and WB was conducted to detect with indicated antibodies. B. PCMV, and PCMV CLK1 plasmids were stably transfected into OVCAR8 and SKOV3 cells. WB was used to determine

CLK1 protein levels. C. CCK8 assay to assess cell viability in pc and oeCLK1 SKOV3 and OVCAR8 cell lines treated with Olaparib for 96 h. D-E. Clonogenic assay to evaluate colony formation efficiency in CLK1 overexpressed SKOV3 (D) and OVCAR8 (E) cells treated with Olaparib for 7–14 days (left). Quantification of colony number (right). F. Representative flow cytometry plots showing apoptosis in CLK1-knockdown ovarian cancer cells (related to quantification in Fig. 3G–H). G–I. Apoptosis analysis by flow cytometry in CLK1-overexpressing SKOV3 (G) and OVCAR8 (H) cells treated with Olaparib (SKOV3: 10 μmol/L; OVCAR8: 5 μmol/L; for 24 h). Representative plots are shown in (I). J. WB analysis of Caspase-3 and cleaved Caspase-3 in control and CLK1-knockdown cells under untreated and Olaparib-treated conditions.

Fig. S4 Clinical analysis of CLK1 expression in OC. Related to Fig. 3

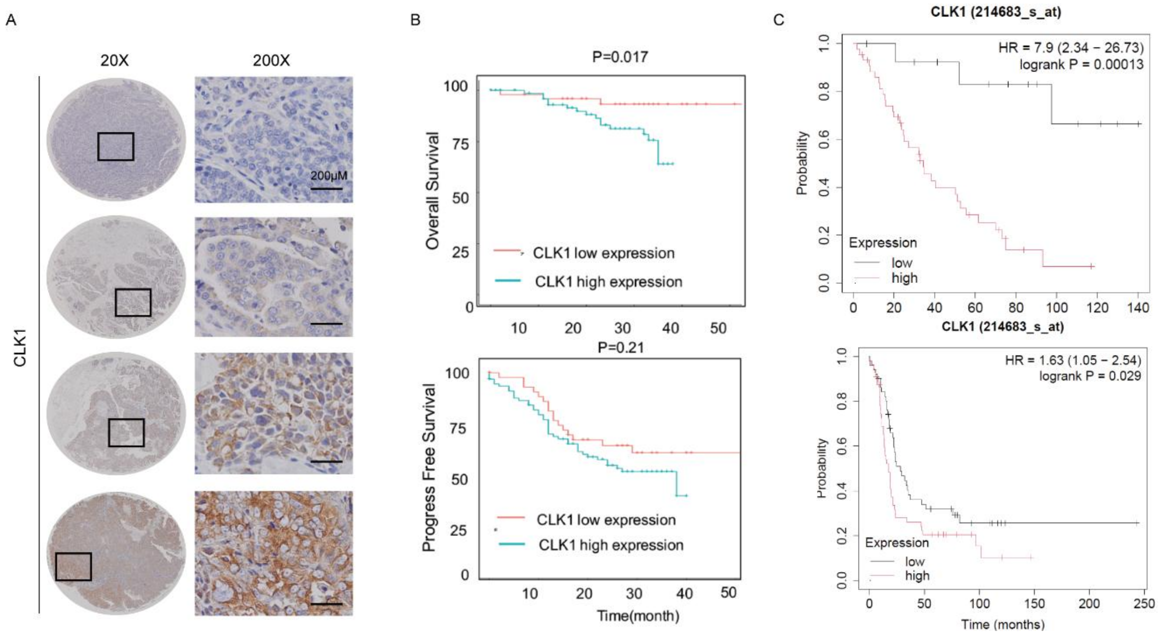

A. Representative IHC staining images of CLK1 at different expression levels in OC tissue microarrays (TMAs). B. Progression-free survival (PFS) and overall survival (OS) curves of OC patients with high or low CLK1 levels in OC TMAs. Log-rank test was used for survival curve comparison between groups. V. OS (up) and PFS (down) of OC patients analyzed by the Kaplan-Meier Plotter with respect to CLK1 expression.

Fig. S5 The knockdown or inhibition of CLK1 activates the DNA damage response pathway. Related to Fig. 4

A

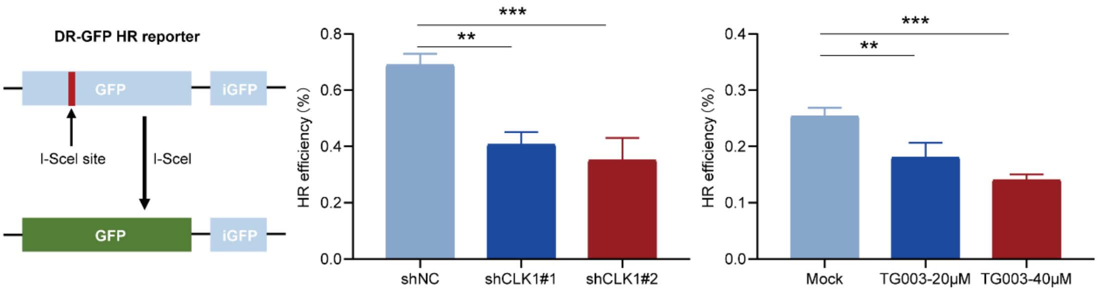

A. HR efficiency after knockdown or inhibition of CLK1 in HeLa cells.

Fig. S6 CLK1 is widely involved in the regulation of DNA damage repair related-proteins, especially ERCC1. Related to Fig. 5

A

GeneID	exonStart	exonEnd	SJC_TG003	SJC_DMSO	PValue	FDR	IncLevel TG003	IncLevel DMSO	IncLevelDifference
ERCC1	45413962	45414034	130,113,136	55,47,42	1.29E-13	9.39E-11	0.516,0.556,0.487	0.717,0.757,0.771	-0.229
ABRAXAS1	83472221	83472288	3,8,3	0,0,1	5.52E-08	8.50E-06	0.764,0.565,0.618	1.0,1.0,0.929	-0.327
POLQ	121473354	121473487	9,8,2	2,0,1	3.16E-05	0.001484955	0.717,0.791,0.919	0.918,1.0,0.966	-0.152
TIPIN	66352128	66352207	0,11,3	0,0,0	4.13E-05	0.001841622	1.0,0.819,0.95	1.0,1.0,1.0	-0.077
SPIDR	47701955	47702015	1,0,11	1,0,0	0.000569154	0.013643349	0.977,1.0,0.83	0.973,1.0,1.0	-0.055
UBE2I	1312346	1312603	6,5,0	11,6,6	0.000618822	0.014487205	0.833,0.859,1.0	0.614,0.786,0.647	0.215
RAD17	69371453	69371557	342,204,212	216,232,227	0.001401775	0.027270903	0.03,0.003,0.0	0.024,0.025,0.052	-0.023
BABAM2	28310081	28310153	34,26,24	30,31,28	0.001821664	0.032912488	0.0,0.0,0.053	0.153,0.0,0.068	-0.056
XRCC3	103712874	103712968	8,0,4	9,13,6	0.001935067	0.034533297	0.729,1.0,0.812	0.471,0.649,0.572	0.283
RBBP8	22957330	22957396	197,235,189	157,113,164	0.002414518	0.040815183	0.004,0.003,0.022	0.022,0.063,0.017	-0.024
UIMC1	177006649	177006794	2,0,0	1,2,4	0.002818123	0.045729205	0.954,1.0,1.0	0.947,0.896,0.798	0.104

A. Inclusion level of the 11 skipping exon genes involved in DNA damage repair.

Fig. S7 CLK1/SRSF5 mediates exon skipping of the ERCC1 gene and contributes to OC sensitivity to PARPi. Related to Fig. 6

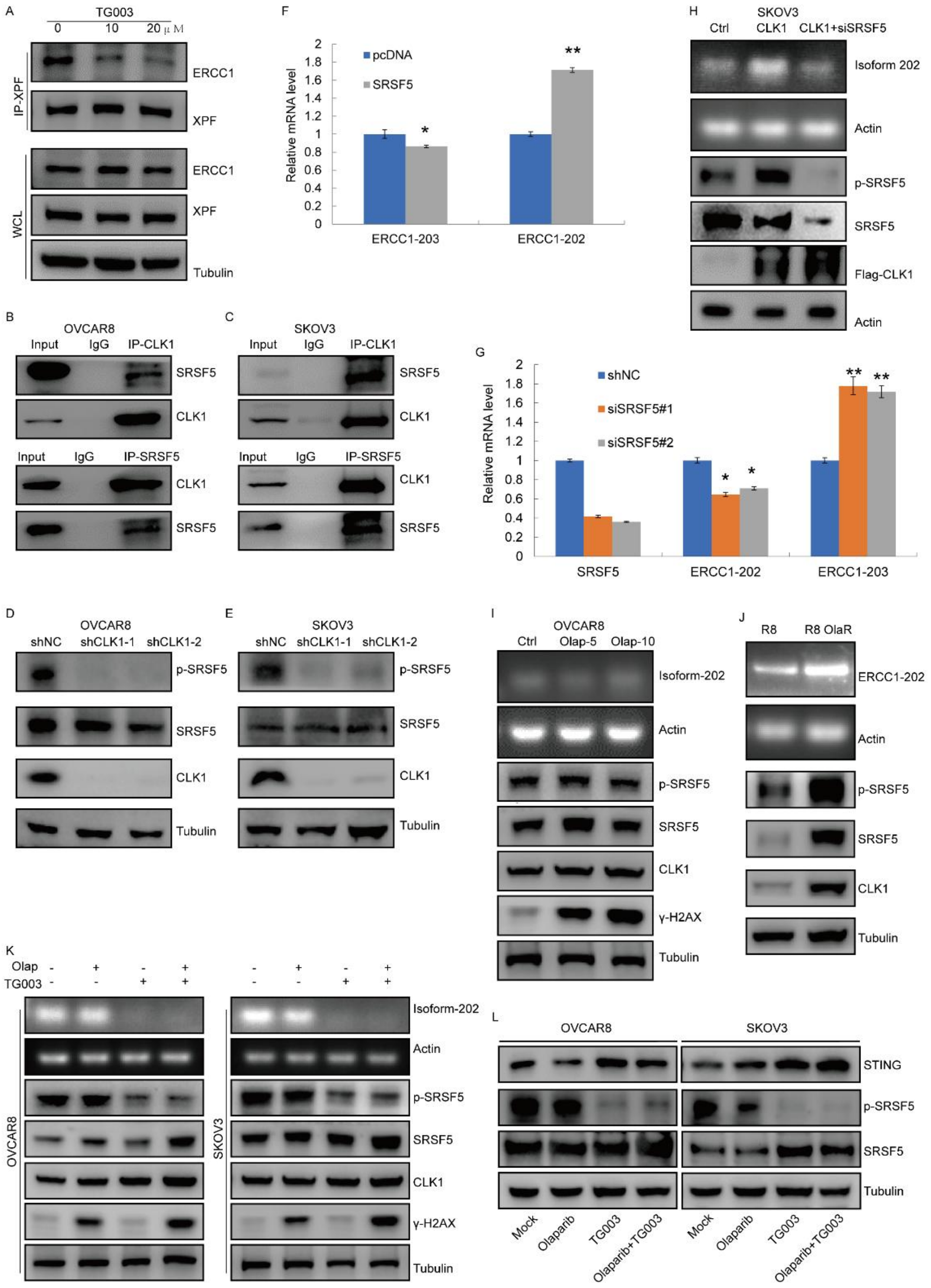

A. Co-IP analysis detecting the interaction between XPF and the ERCC1-202 isoform in OVCAR8 cells treated with TG003 for 24h. B-C. Co-IP analysis detecting the interaction between CLK1 and SRSF5 in OVCAR8 (B) and SKOV3 (C) cells. D-E. WB analysis detected the phosphorylation of SRSF5 after CLK1 knockdown in OVCAR8 (D) and SKOV3 (E) cells. F-G. RT-qPCR analysis of mRNA expression levels of ERCC1-202 and ERCC1-203 isoforms after SRSF5 overexpression or knockdown in OVCAR8 cells. H. ERCC1-202 isoform expression in SKOV3 control, CLK1-overexpressing, and CLK1-overexpressing/SRSF5-knockdown cells. I-J. Analysis of ERCC1-202, CLK1, and p-SRSF5 expression levels in OVCAR8 cells upon exposure to PARPi (I) and in established PARPi-resistant cells (J). K. Analysis

of ERCC1-202, CLK1, and p-SRSF5 expression levels in OVCAR8 and SKOV3 cells with Olaparib and TG003 alone or in combination respectively. L. WB conducted in OVCAR8 and SKOV3 cells treated with Olaparib and TG003 alone or in combination respectively.
